# Amylin at the interface between metabolic and neurodegenerative disorders

**DOI:** 10.3389/fnins.2015.00216

**Published:** 2015-06-16

**Authors:** Thomas A. Lutz, Urs Meyer

**Affiliations:** ^1^Institute of Veterinary Physiology, University of ZurichZurich, Switzerland; ^2^Zurich Center of Integrative Human Physiology, University of ZurichZurich, Switzerland; ^3^Institute of Veterinary Pharmacology and Toxicology, University of ZurichZurich, Switzerland

**Keywords:** amylin, Alzheimer's disease (AD), β-amyloid, dementia, hyperglycemia, insulin, obesity, type-2 diabetes

## Abstract

The pancreatic peptide amylin is best known for its role as a satiation hormone in the control of food intake and as the major component of islet amyloid deposits in the pancreatic islets of patients with type 2 diabetes mellitus (T2DM). Epidemiological studies have established a clear association between metabolic and neurodegenerative disorders in general, and between T2DM and Alzheimer's disease (AD) in particular. Here, we discuss that amylin may be an important player acting at the interface between these metabolic and neurodegenerative disorders. Abnormal amylin production is a hallmark peripheral pathology both in the early (pre-diabetic) and late phases of T2DM, where hyperamylinemic (early phase) and hypoamylinemic (late phase) conditions coincide with hyper- and hypo-insulinemia, respectively. Moreover, there are notable biochemical similarities between amylin and β-amyloids (Aβ), which are both prone to amyloid plaque formation and to cytotoxic effects. Amylin's propensity to form amyloid plaques is not restricted to pancreatic islet cells, but readily extends to the CNS, where it has been found to co-localize with Aβ plaques in at least a subset of AD patients. Hence, amylin may constitute a “second amyloid” in neurodegenerative disorders such as AD. We further argue that hyperamylinemic conditions may be more relevant for the early processes of amyloid formation in the CNS, whereas hypoamylinemic conditions may be more strongly associated with late stages of central amyloid pathologies. Advancing our understanding of these temporal relationships may help to establish amylin-based interventions in the treatment of AD and other neurodegenerative disorders with metabolic comorbidities.

## Introduction

A growing number of individuals suffer from aging-associated neurological and cognitive dysfunctions that are characterized by progressive neurodegeneration and dementia. The global prevalence of dementia is currently estimated to be as high as 30–40 million, and is predicted to quadruple by the year 2050 (Reitz et al., 2011; Hurd et al., 2013; Reitz and Mayeux, 2014). The most common form of aging-associated dementia is late-onset or sporadic Alzheimer's disease (AD) (Hampel et al., 2011; Reitz et al., 2011), a neurodegenerative disorder affecting multiple cognitive and behavioral functions. AD patients typically show progressive cognitive decline along with the concomitant appearance of several neuropathological hallmarks, including amyloid plaque deposition, neurofibrillary tangle formation, vascular malfunction, and synaptic loss (Ferrer, 2012). Several other pathological processes have been identified in the early stages of the disease, which in turn may play a major etiopathological role in sporadic AD. These include increased oxidative stress, neuroinflammation, and impairments in brain glucose uptake and metabolism (Eikelenboom et al., 2010; Bassil et al., 2014; Chen and Zhong, 2014; Morales et al., 2014). Hence, sporadic AD likely represents a multifactorial disease caused by a multitude of synergistically interacting pathological changes, resulting in severe cognitive impairments and extensive brain tissue atrophy (McDonald, 2002).

In parallel to the global increase in neurodegenerative disorders, there is a rising prevalence of metabolic diseases, most notably obesity and type-2 diabetes mellitus (T2DM) (Ginter and Simko, 2012a,b). Obesity is typically referred to as a medical condition, in which excess body fat has accumulated to the extent that the body mass index (BMI) exceeds 30 kg/m^2^ (Chiang et al., 2011). It is thought to be one of the primary causes of T2DM (Chiang et al., 2011; Ginter and Simko, 2012a,b). Depending on its clinical course and disease stage, T2DM involves altered glycemic regulation and hyperglycemia, resistance to insulin actions, inadequate insulin secretion from pancreatic β-cells, as well as progressive loss of pancreatic β-cells which is paralleled by deposition of islet amyloid polypeptide, or amylin, as pancreatic amyloid (Kahn et al., 2014).

Besides their negative effects on body homeostasis, peripheral metabolic diseases also appear to play an important role in aging-associated cognitive impairments and neurodegenerative disorders such as AD (de la Monte and Tong, 2014; Ríos et al., 2014). Epidemiological studies have repeatedly shown a significant association between (midlife) obesity and late-onset dementias (Yaffe, 2007; Arnoldussen et al., 2014; Kiliaan et al., 2014; Emmerzaal et al., 2015). Likewise, patients with T2DM are 2- to 5-times more likely to develop AD compared to non-diabetic individuals (Ott et al., 1996; Li and Hölscher, 2007; Butterfield et al., 2014; Desai et al., 2014). Additional support for these epidemiological associations can be derived from investigations in animal models showing that diet-induced obesity and T2DM impair behavioral and cognitive functions relevant to human dementia (Greenwood and Winocur, 2005; Winocur and Greenwood, 2005; Morris and Tangney, 2014) and exacerbate neuropathological and cognitive deficits in genetic mouse models of AD (Leboucher et al., 2013; Vandal et al., 2014). Based on these findings, it has been proposed that AD and related neurodegenerative disorders may represent a form of “type-3 diabetes.” According to this hypothesis, impaired brain insulin and insulin-like growth factor (IGF) signaling, or “brain insulin resistance,” may trigger and promote neurodegenerative processes via multiple interrelated pathological mechanisms, including attenuation of brain glucose uptake and subsequent emergence of central hypometabolism, mitochondrial dysfunction and oxidative stress, cerebro-vascular disruption, as well as imbalances in neuroinflammatory reactions (reviewed in de la Monte and Wands, 2008; de la Monte, 2012; Bassil et al., 2014; Sebastião et al., 2014). At the same time, some of the AD-associated neuropathologies themselves, especially extracellular amyloid plaques, may promote brain insulin resistance (de la Monte, 2012). Hence, there could be a positive feedback loop between brain insulin resistance and amyloid plaque formation, which together may facilitate progressive neurodegeneration and cognitive decline (de la Monte, 2012). This hypothesis is also supported by recent preclinical investigations and clinical trials demonstrating beneficial effects of compounds with anti-diabetic properties on attenuating or normalizing cognitive impairments and AD-related neuropathologies (for review, see Bassil et al., 2014; Sebastião et al., 2014).

In the present article, we discuss the potential role of the pancreatic peptide amylin, also called islet amyloid polypeptide (IAPP), as a link between metabolic and neurodegenerative disorders in general, and between T2DM and AD in particular. Amylin is best known for its role as a satiation hormone in the control of food intake (Lutz, 2005, 2009, 2010) and as the major component of amyloid deposits in pancreatic islets of patients with T2DM and of cats (Lutz and Rand, 1993; Westermark et al., 2000; Osto et al., 2013). Because of its propensity to form amyloid deposits in susceptible species like primates and cats, amylin shares several pathological features with β-amyloids (Aβ), a family of amyloidogenic peptides most frequently implicated in the neuropathology of AD (Karran et al., 2011; Seeman and Seeman, 2011). Here, we discuss these similarities following a concise introduction in amylin's main physiological roles. Particular emphasis is then placed on the potential mechanisms of amylin and Aβ amyloidogenesis, and on the recent findings directly implicating amylin amyloids in the neuropathology of AD.

## Physiological roles and actions of amylin

### Amylin acts as a satiation signal

Amylin is a pancreatic β-cell hormone co-released with insulin in response to food intake (Kahn et al., 1990). It reduces eating, gastric acid secretion, limits the rate of gastric emptying, and diminishes pancreatic glucagon secretion (Lutz, 2010). One of the best-studied functions of amylin relates its role as a satiation signal: via regulating meal size (Lutz et al., 1995b; Reidelberger et al., 2002; Mollet et al., 2004), it contributes to meal termination akin to other typical satiating hormones such as cholecystokinin (CCK) (Young and Denaro, 1998; Lutz, 2010). The anorectic actions of amylin represent physiological effects as eating leads to a rapid increase in circulating amylin levels, and the administration of exogenous amylin reduces eating within minutes after application. Further, amylin's capacity to reduce meal size is dose dependent and is effectively and specifically blocked by amylin antagonists like AC187 (Mollet et al., 2004; Reidelberger et al., 2004). Importantly, amylin and its synthetic analogs reduce meal size without producing signs of conditioned taste aversion or visceral illness (Lutz et al., 1995b; Rushing et al., 2002; Mack et al., 2007). Overall, these data indicate that amylin acts as a specific and physiological satiating hormone that mediates its short-term effects on food intake mainly via reducing meal sizes.

### Amylin acts as adiposity signal

The concept of “homeostatic eating controls” classically distinguishes between adiposity (“tonic”) signals and meal-associated (“episodic”) signals. Adiposity signals are secreted by adipose tissue (leptin) or the pancreas (insulin, amylin) in proportion to body adiposity and are thought to reduce eating by enhancing the effects of satiation signals. For example, amylin enhances the satiating effects of cholecystokinin (CCK) (Bhavsar et al., 1998; Mollet et al., 2003). A number of other findings support a role for amylin as an adiposity signal. For example, basal plasma levels of amylin are higher in obese relative to lean rats (Pieber et al., 1994), and high-fat fed obese rats have higher baseline amylin levels compared to lean controls (Boyle and Lutz, 2011; Boyle et al., 2011). Moreover, chronic peripheral (Arnelo et al., 1996; Lutz et al., 2001) or central (Rushing et al., 2002) administration of amylin decreases body weight and fat gain, whereas treatment with amylin antagonists increases body adiposity (Rushing et al., 2001). Together, these data suggest that central amylin, like leptin or insulin, may encode a certain level of body weight, and therefore, may contribute to the relative constancy of body weight throughout adult life.

### Amylin and energy expenditure

The physiological roles and actions of amylin have been further explored using pair-feeding research designs in rats, in which the amount of food available for vehicle-treated animals was determined by the amount of food ingested by amylin-treated animals. These studies showed that the reduction in body fat is greater in rats chronically treated with exogenous amylin as compared to pair-fed controls (Isaksson et al., 2005; Roth et al., 2006; Mack et al., 2007). These findings indicate that amylin not only reduces food intake but also increases energy expenditure (Isaksson et al., 2005; Wielinga et al., 2007, 2010). Again, these dual effects of amylin are similar to those induced by leptin, which is known to influence energy balance through concomitant effects on eating and an energy expenditure (Friedman, 1997; Schwartz et al., 2000, 2003). Increases in energy expenditure can also been seen in rats after acute central infusion of amylin into discrete brain areas, including the third cerebral ventricle or area postrema (AP) (Osaka et al., 2008; Wielinga et al., 2010).

Two recent studies provided novel mechanistic insights into amylin's effect on energy metabolism (Zhang et al., 2011; Fernandes-Santos et al., 2013). Similar to humans, total energy expenditure in rodents partially depends on the thermogenic activity of the brown adipose tissue (BAT), which in turn is controlled by the sympathetic nervous system (Nedergaard and Cannon, 2014). BAT thermogenic activity is increased by amylin through actions involving the sympathetic nervous system, and these effects are enhanced in transgenic mice that overexpress the receptor activity-modifying protein 1 (RAMP1) of the amylin receptor complex. More specifically, transgenic mice with neuronal overexpression of RAMP1 show a potentiated metabolic response to exogenous amylin treatment and display a number of basal metabolic changes, including reduced body weight and fat mass, increased energy expenditure and body temperature, and hypophagia. It has been further shown that the effects of RAMP1 overexpression on energy expenditure are mediated by an increased sympathetic tone in efferents innervating BAT (Zhang et al., 2011; Fernandes-Santos et al., 2013).

## Central mechanisms underlying the actions of amylin

Amylin receptor autoradiography studies demonstrate that several brain areas have high affinity binding sites for amylin (Sexton et al., 1994). Amylin binding is particularly strong in the circumventricular organs such as the subfornical organ (SFO) and AP. A large number of studies indicate that the AP plays an important role in mediating the physiological effects of peripheral amylin. Amylin appears to activate AP neurons by direct humoral actions, whilst vagal or non-vagal afferents do not seem to be required in these processes (Edwards et al., 1998; Lutz et al., 1995a, 1998a,b; Wickbom et al., 2008; Mack et al., 2010; Braegger et al., 2014). Local amylin administration into the AP recapitulates the effects of peripheral amylin, and AP-directed infusion of the amylin antagonist AC187 exerts the opposite effects. Furthermore, local administration of AC187 into the AP has been shown to abolish the eating inhibitory effect of peripheral amylin (Mollet et al., 2004), suggesting that central actions in the AP are necessary for mediating the anorectic effects of peripheral amylin.

Amylin-induced activation of AP neurons readily induces a number of neuronal downstream effects, including stimulation of dopamine-beta-hydroxylase (DBH) and subsequent increase in noradrenaline synthesis and release in noradrenergic output areas (Potes et al., 2010). In addition to its effects on noradrenergic neurons, amylin may further target other neurotransmitter systems in the AP, including presynaptic glutamatergic terminals innervating AP neurons (Fukuda et al., 2013). The functional contribution of these glutamatergic terminals, however, remains unknown with regards to amylin's effects on food intake, metabolism, and other physiological processes.

Stimulation of AP neurons by peripheral amylin likely leads to the activation of a neuroaxis that projects rostrally to the forebrain and includes the nucleus tractus solitarii (NTS), lateral parabrachial nuclus (LPB), and central amygdala (CeA) (Rowland et al., 1997; Riediger et al., 2004). In addition to its stimulating effects on these brain regions, which most likely follows primary AP activation (Riediger et al., 2004), amylin further seems to activate key areas of the mesolimbic dopamine system, including the ventral tegmental areas (VTA) and nucleus accumbens (NAc). These two regions display high affinity amylin binding sites and seem to contain virtually all components of the amylin receptor complex (Mietlicki-Baase et al., 2013; Mietlicki-Baase and Hayes, 2014). The mesolimbic dopamine system has long been recognized to play a crucial role in reward processing including eating associated rewards (Berridge and Robinson, 1998; Volkow et al., 2013). With respect to the latter, it has been suggested that pathological conditions of overeating may represent a compensation for decreased activity of the mesolimbic and nigrostriatal dopamine circuits (Wang et al., 2001). This hypothesis is consistent with the notion that the rewarding properties of food can stimulate feeding behavior even when energy requirements have been met, thereby contributing to excessive weight gain and obesity (Kenny, 2011).

In this context, it is interesting that direct local administration of the amylin receptor and calcitonin receptor agonist salmon calcitonin into the VTA of rats has been found to reduce the intake of standard rodent chow diet and of palatable sucrose. These anorectic effects appeared to result from reductions in meal size, suggesting that activation of VTA neurons by salmon calcitonin may modulate the reward value of food, thereby reducing the size of a meal. On the contrary, administration of the amylin antagonist AC187 into the VTA increased eating, and intra-accumbens injection of AC187 attenuated the anorectic effects of peripheral treatment with salmon calcitonin (Mietlicki-Baase et al., 2013); local knockdown of the calcitonin receptor (CTR)—the core component of functional amylin receptors (Bailey et al., 2012)—in the VTA also resulted in hyperphagia (Mietlicki-Baase et al., 2015). Further, salmon calcitonin administered to the VTA resulted in reduced phasic dopamine release into the NAc core, and blockade of dopamine D1 and D2 receptors in the NAc core blunted salmon calcitonin's eating inhibitory effect (Mietlicki-Baase et al., 2015). Finally, the NAc shell may also be directly responsive to amylin receptor stimulation because low doses of amylin given into the NAc shell reduced eating in rats; amylin also inhibited the eating response induced by opioid receptor activation indicating that amylin signaling may negatively modulate opioid reward driven eating (Baisley and Baldo, 2014).

Together, these findings indicate that peripheral amylin may, in addition to its actions on the AP and interconnected areas, exert a direct influence on VTA or NAc neurons and induce physiologically relevant effects on satiation and food reward. It will be important to test in future studies whether the observed effects via the VTA or NAc do depend on a primary action of amylin in these areas or perhaps still in the AP. It has been shown that the rat amylin-1 receptor (Bailey et al., 2012) is activated equally by amylin but also the neurotransmitter calcitonin gene-related peptide (CGRP), and, importantly, that the effects of both peptides at the amylin-1 receptor are blocked by AC187. Hence, it is possible that primary activation of AP neurons may trigger CGRP release in various brain areas, including the VTA or the NAc, to explain the observations discussed above.

## Amylin amyloid formation

The aforementioned physiological effects of amylin all require solubility of the monomeric hormone so that it can effectively reach the target organs and cells upon release by pancreatic β-cells. Under certain pathological conditions such as T2DM, however, amylin can self-aggregate and eventually form insoluble amylin amyloid plaques. As described in detail in the subsequent sections, this effect is only relevant in few species, including humans, other primates and cats when amylin amyloid loses its common physiological actions, but instead, induces toxic effects on both peripheral and central organs and cells.

### Mechanisms of amyloidogenesis

The secreted and biologically active form of amylin contains 37 amino acids. It is initially expressed as a pre-pro-protein containing 89 amino acids, which includes the actual signal peptide domain (22 acids) and two short flanking peptides (Höppener et al., 1994; Marzban et al., 2004, 2005; Wang et al., 2011). Similar to pro-insulin, pre-pro-amylin is processed to pro-amylin in the endoplasmic reticulum (ER), during which the signal peptide and flanking peptides are cleaved from the rest. The resulting pro-amylin is then further processed by proteolysis and posttranslational modifications in the Golgi apparatus, where a disulfide bond between cysteine residues 2 and 7 is formed. The biologically active amylin is then stored in the halo region of the secretory granules of the β-cell and is co-secreted with insulin (Westermark et al., 1996).

The amino acid sequence of amylin is strongly conserved among mammalian species. Formation of amylin amyloids, however, can only emerge in the pancreatic islets of humans, primates, and cats, but not in rodents. The reason for this species differences is that only the former contain an amyloidogenic region within their amino acid sequence that is essential for amyloid formation and toxicity (Betsholtz et al., 1989; Höppener et al., 1994; Moriarty and Raleigh, 1999; Matveyenko and Butler, 2006). In the case of amylin, amyloidogenesis seems to be associated with the heterogeneity of the amino acid residues 20–29 of the amylin sequence. In species where pancreatic amyloid deposition does not occur (e.g., mouse or rat), one or more proline residues can be found in this region. Hence, it is believed that the presence of proline prevents the formation of ordered secondary structures such as the β-sheet conformation, which is an essential prerequisite for amyloidogenesis (Betsholtz et al., 1989; Höppener et al., 1994; Moriarty and Raleigh, 1999; Matveyenko and Butler, 2006).

Under physiological conditions, monomeric amylin is soluble and natively unfolded. It is believed that amylin is protected from aggregation under these conditions by interactions with other components of the secretory vesicles, including insulin, pro-insulin, or their processing intermediates (Westermark et al., 1996). It seems that a relative increase in the concentration of amylin within the halo region of pancreatic islet cells can initiate their aggregation and formation of fibrils under certain conditions (Paulsson et al., 2006). When fibril formation begins, the molecules in β-sheet conformation are bound to each other, predominantly by hydrogen bounds. The β-sheet conformation typically forms thin and stable layers, in which the β-strands are oriented perpendicular to the fibril axis.

Interestingly, amylin's amyloidogenic properties are not restricted to mature amylin but further extend to pro-amylin and its intermediates (Westermark et al., 2000; Paulsson and Westermark, 2005; Paulsson et al., 2006). In fact, the latter may be the starting point of oligomerization and amylin aggregation within the pancreatic islets (Westermark et al., 2000; Jaikaran and Clark, 2001; Jaikaran et al., 2001; Paulsson et al., 2006). It should be noted, however, that no precise localization of the aggregates within the cells has been demonstrated yet. In studies using baboons, amyloid deposits were observed in the cytoplasm of β-cells as well as bound to the outer β-cell membrane (Guardado-Mendoza et al., 2009). In other studies, where islets from diabetic patients were transplanted into nude mice or cultured *in vitro*, amylin-derived amyloid was found to form an intracellular network, suggesting its presence in the ER, Golgi apparatus and secretory vesicles (Westermark et al., 1995a,b; Yagui et al., 1995; Paulsson et al., 2006). Recent evidence further suggests that the early processes of amyloidogenesis from amylin depend on pH, which can markedly affect the electrostatic interactions required for β-sheet formation and amylin fibrillization (Li et al., 2013; Jha et al., 2014).

Abnormalities in the processing of amylin and its deposition as amyloid in the islets may contribute to the progressive loss of pancreatic β-cells, which is a pathological hallmark of T2DM. Indeed, islet amyloid formation is a striking abnormality of human T2DM and can be found in more than 90% of T2DM patients (Clark and Nilsson, 2004). Similarly, islet amyloid is a very prominent feature in diabetic cats (Lutz and Rand, 1993; Osto et al., 2013). Even though the association between amylin amyloid deposition and pancreatic β-cell loss in T2DM seems robust, the underlying etiopathological processes remain to be determined. According to one hypothesis, prolonged hyperglycemia may lead to a higher concentration of pro-amylin within the β-cells (Hou et al., 1999), which in turn may create a core for amylin aggregation and ultimately cause β-cell loss and deposition of extracellular amyloid (Paulsson and Westermark, 2005). An alternative (but mutually not exclusive) mechanism for amylin aggregation may include increased retention of amylin in β-cell granules as a result of increased sympathetic stimulation (Watve et al., 2014). In addition, high levels of circulating free fatty acids, as observed in pre-diabetic patients or patients with overt T2DM, has been implicated as possible mechanism facilitating amyloid formation (Li and Hölscher, 2007).

### Pathological effects of amylin deposits on pancreatic β-cells

Islet amyloid formation plays a key role in β-cell apoptosis and dysfunction as seen in T2DM (Westermark et al., 2000, 2011; Paulsson and Westermark, 2005; Höppener and Lips, 2006; Jurgens et al., 2011). As discussed in the subsequent sections, it is believed that the toxic effects induced by amylin amyloid deposits depend on a combination of mechanisms that act both intra- and extra-cellulary.

One of the prominent features associated with amylin-related β-cell toxicity is ER stress (Preston et al., 2009). Peripheral insulin resistance triggers β-cells to produce more insulin in parallel with an increased synthesis of amylin. As these proteins are destined for exocytosis, they are transported through the ER and trans-Golgi complex and stored in secretory granules before secretion. ER stress is characterized by the unfolded protein response (UPR), which is induced to cope with an increase in protein synthesis or obstruction in the ER-Golgi transport (Preston et al., 2009). UPR involves the upregulation of ER-located chaperones to assist in the folding of proteins that are prone to aggregation. Under homeostatic conditions, misfolded proteins are normally transported to the ubiquitin-proteasome system for degradation, thus preventing protein aggregation and amyloidogenesis. Failure of such homestatic responses typically leads to induction of cell apoptosis (Oyadomari et al., 2002; Marciniak and Ron, 2006; Davenport et al., 2008). Such processes may be operational especially during the early phase of T2DM, during which amylin and insulin syntheses are markedly increased as a consequence of insulin resistance, leading to non-homeostatic UPR and eventually β-cell apoptosis.

Abnormally enhanced accumulation of fibrillar material in the halo region of secretory vesicles may also lead to a disruption of the vesicles and result in the release of fibrillar material into the cytosol. Because the proteasome pathway can only degrade misfolded single proteins but not their aggregates, a cellular process commonly referred to as “autophagy” dissolves the latter. It is a well-preserved catabolic process that is activated to degrade and recycle misfolded proteins and excess or defective organelles (Stienstra et al., 2014). Under non-pathological conditions, autophagosomes typically fuse with lysosomes. This causes the formation of autophagolysosomes, where misfolded proteins or excess/defective organelles can be degraded efficiently. Under pathological conditions such as the early phase of T2DM, however, the formation of autophagolysosomes is attenuated, which in turn causes the accumulation of material in autophagic vacuoles without being further processed (Stienstra et al., 2014). Accumulation of autophagic vacuoles may disturb intracellular cell transport and induce a cascade of intracellular toxic effects (Marciniak et al., 2006; Marciniak and Ron, 2006; Davenport et al., 2008; Stienstra et al., 2014). It has been suggested that pathological processes involving accumulation of autophagic vacuoles may also play a role in pancreatic β-cell toxicity (Bursch et al., 2000; Stienstra et al., 2014).

The overexpression of pro-amylin or amylin during the early phases of T2DM may further lead to the formation of toxic oligomers, which enter the cytosol and disrupt the membrane of organelles such as mitochondria. The disruption of mitochondria function may then result in increased cellular oxidative stress and the formation of reactive oxygen species (ROS). Increased ROS levels may ultimately lead to significant damage to cell structures and cause cell loss (Parks et al., 2001; Scherz-Shouval and Elazar, 2007; Gurlo et al., 2010). Finally, it appears that increased amylin production can activate the inflammasome, which is a multiprotein component of the innate immune system producing the inflammatory cytokine interleukine-1β (IL-1β). Increased inflammatory processes in general, and overproduction of IL-1β in particular, may represent another important mechanism by which amylin aggregation can cause β-cell death (Masters et al., 2010; Donath and Shoelson, 2011; Westwell-Roper et al., 2011; Donath et al., 2013).

## Amylin amyloids: a comparison with the formation and pathological consequences of Aβ amyloids

The propensity of amylin to form pancreatic amyloids and their cytotoxic consequences are remarkably similar to β-amyloids (Aβ), which are prone to self-aggregate in the CNS following enzymatic processing of the amyloid precursor protein (APP). The type-I transmembrane glycoprotein APP is one of the most abundant proteins in the human CNS and is ubiquitously expressed in the plasma membrane and in several organelles, such as the ER, Golgi apparatus, and mitochondria (van der Kant and Goldstein, 2015).

In general, APP can be processed by two distinct and mutually exclusive pathways, namely the secretory pathway and the amyloidogenic pathway (Karran et al., 2011). Whilst the former leads to soluble fragments, the latter creates proteolytic products that are prone to self-aggregation. In the secretory pathway, APP is first cleaved by α-secretase, which results in the release of a soluble N-terminal fragment (sAPPα) and a C-terminal fragment (C83). The C-terminal fragment is then further processed by another cleaving enzyme (γ-secretase) to generate a smaller and soluble C-terminal fragment of approximately 3 kDa (C3). Notably, the cleavage of APP by α-secretase occurs within the sequence of amino acids that would form the Aβ peptide, and therefore, this proteolytic pathway is crucial and readily prevents the formation of self-aggregating amyloid peptides (Karran et al., 2011). In the amyloidogenic pathway, however, APP is cleaved by β-secretase, which releases a small N-terminal fragment (sAPPβ) and a long C-terminal fragment (C99). The latter contains the full amyloidogenic sequence of amino acids and is further processed by γ-secretase to yield amyloid-β (Aβ) peptides. These are then released as monomers and eventually aggregate progressively into oligomers and finally into amyloid plaques.

Aβ oligomers are considered the most neurotoxic forms of all amyloid species. As extensively reviewed elsewhere (Hardy and Selkoe, 2002; Karran et al., 2011; van der Kant and Goldstein, 2015), they interact with neuronal and glial cells to cause mitochondrial dysfunction and oxidative stress, impairments of intracellular signaling pathways and synaptic plasticity, and eventually neuronal apoptosis. It is believed that these mechanisms can give rise to a positive feedback loop that facilitates the production of Aβ peptides through stimulation of the amyloidogenic pathway (Hardy and Selkoe, 2002; Karran et al., 2011; van der Kant and Goldstein, 2015). Hence, the amyloidogenic pathway seems to be more active under pathological conditions, whereas APP is preferentially processed via the secretory pathway under physiological conditions.

The maintenance of physiological conditions is further dependent on the equilibrium between the production of (soluble) Aβ peptides and their clearance from the brain (Ueno et al., 2014; Baranello et al., 2015). Even though the precise mechanisms by which Aβ peptides are cleared from the CNS remain to be identified, two proteins seem to be pivotal for this process, namely apolipoprotein E (APOE) and the insulin-degrading enzyme (IDE) (Baranello et al., 2015). It has been proposed that these proteins may bind to the Aβ peptide, thereby preventing aggregation and promoting clearance from the brain. Some of the known genetic risk factors of AD, including polymorphisms in the ε 4 allele of APOE, may predisposes individuals to impaired clearance of Aβ peptides from the CNS, and thus facilitate the formation of Aβ plaques (Baranello et al., 2015). Aβ clearance can also be disrupted by several metabolic disturbances in general, and by those associated with T2DM in particular. Indeed, T2DM has been linked to IDE deficiency, which in turn can facilitate the aggregation of amyloidogenic proteins (Steneberg et al., 2013), including Aβ (Farris et al., 2003) and amylin-derived amyloid (Bennett et al., 2003).

These associations have several important implications. First, the effects of IDE hypofunction on Aβ plaque formation may represent a crucial pathological link between metabolic disorders such as T2DM and increased risk of AD. Second, IDE deficiency may not only be a driving force for amylin oligomerization and cytotoxicity in pancreatic islet cells, but it may further facilitate the formation of amylin amyloid in the CNS. In support of the latter hypothesis and in contrast to the original belief that amylin-derived amyloid occurs exclusively in pancreatic islets, amylin oligomers and plaques were recently identified in the temporal lobe gray matter from patients with T2DM (Jackson et al., 2013). Moreover, similar amylin deposits were also detected in brain parenchyma of patients with late onset AD, and these amylin amyloids co-localized with Aβ oligomers and plaques (Jackson et al., 2013). These observations suggested for the first time that metabolic disorders such as T2DM and perhaps also aging might similarly promote the accumulation of amylin amyloid in the CNS. Based on these findings, it has been hypothesized that amylin could constitute a “second amyloid” in AD (Jackson et al., 2013).

More recent studies indicated that amylin and Aβ co-localize in plaques of AD brains, and that amyloid deposits consisting of both amylin and Aβ are also present in blood vessel walls (Oskarsson et al., 2015). While it is not yet clear whether the amylin that forms deposits in the brain is produced locally or is rather secreted by the pancreas, it is of note that intravenous injection of preformed amylin fibrils did enhance local amyloid deposition in the pancreas, hence a systemic origin is plausible (Oskarsson et al., 2015; see also next section).

## How is altered amylin secretion linked to central amyloid formation?

Despite the existing evidence for amylin oligomerization in the brains of T2DM and AD patients (Jackson et al., 2013), it remains controversial whether this neuropathological effect can be related to, or even induced by, alterations in peripheral amylin homeostasis. Some researchers suggest that the primary mechanism leading to amylin deposition in the brains of patients with T2DM and AD may be hyperamylinemia, i.e., a pathological condition of chronic hypersecretion of amylin by pancreatic β-cells (Srodulski et al., 2014). Hyperamylinemia is frequently observed in individuals with obesity or pre-diabetic insulin resistance (Johnson et al., 1989; Enoki et al., 1992) and often coincides with hyperinsulinemia (Westermark et al., 2011). Furthermore, genetically enforced overexpression of amylin in rodents transgenic for the human form of amylin leads to amylin oligomerization and amyloid formation in pancreatic β-cells, β-cell apoptosis, and eventual development of T2DM (Matveyenko and Butler, 2006; Huang et al., 2007). Another cornerstone of the “hyperamylinemia hypothesis” of central amylin deposition is that peripherally produced amylin readily crosses the blood-brain barrier (Banks and Kastin, 1998). Hence, increased peripheral secretion and central uptake of amylin may facilitate amylin oligomerization and eventually amyloid formation in the brain, thereby disrupting the integrity of CNS structures and functions. Support for this hypothesis has recently been obtained in a “humanized” rat model of hyperamylinemia (Srodulski et al., 2014): Rats with pancreatic overexpression of human amylin showed marked amylin amyloid formation, an effect that was paralleled by neuroinflammatory processes such as clustering of activated microglia in areas of amylin infiltration. These neuropathological changes were further associated with the development of behavioral and cognitive deficits, including impaired recognition memory and motor learning (Srodulski et al., 2014). These findings suggest that hypersecretion of amyloidogenic forms of amylin might indeed represent a pathological link between amylin amyloid formation and the development of neurological deficits.

The “hyperamylinemia hypothesis,” however, may only hold true for central amylin deposition, but not for the accumulation and oligomerization of other amyloidogenic peptides such as Aβ. Indeed, in the Tg2576 genetic mouse model of AD, which harbors a mutant form of APP that is prone to marked Aβ plaque formation, amylin plasma levels were not significantly elevated at an age when mice exhibited clear signs of T2DM (Fawver et al., 2014). These findings argued against the hypothesis that altered systemic amylin levels might promote cross-seeding of Aβ in the CNS, despite the fact that amylin crosses the blood-brain barrier and efficiently facilitates Aβ oligomerization *in vitro* (Fawver et al., 2014). A limitation of these findings, and the interpretations thereof, is that transgenic Tg2576 mice only harbor mutations that facilitate Aβ plaque formation, but not amylin plaque formation. Indeed, unlike in humans, primates and cats, rodent amylin *per se* is not amyloidogenic (Betsholtz et al., 1989; Höppener et al., 1994; Moriarty and Raleigh, 1999; Matveyenko and Butler, 2006). Therefore, the missing association between systemic amylin and Aβ plaque formation in Tg2576 mice may be explained by the native incapacity of mouse amylin to form amylin plaques, which in turn may limit the capacity of amylin to cross-seed Aβ in the CNS. In this respect, mice clearly differ from humans.

Adding to these controversies, recent research even suggests that reduced, rather than increased, systemic levels of amylin may facilitate the development of AD-like pathology. For example, Zhu et al. (2015) revealed that the augmentation of endogenous amylin by chronic systemic administration of non-amyloidogenic amylin, or its clinical analog pramlintide, has beneficial effects against neuropathological and cognitive deficits in a mouse model of AD. Specifically, peripheral treatment with amylin or pramlintide reduced the number of Aβ plaques and improved the deficits in short- and long-term memory in PP/PS1 double transgenic mice that harbor five familial genetic mutations implicated in AD (Zhu et al., 2015). Zhu et al. (2015) further demonstrated that peripheral amylin or pramlintide treatment similarly increased the concentrations of Aβ_1−42_ in cerebral spinal fluid (CSF) and serum, suggesting that the augmentation of non-amyloidogenic amylin might facilitate the clearance of Aβ peptides from the CNS. Similar beneficial effects have been found by another research group, who found that chronic administration of the amylin analog pramlintide, which lacks amyloidogenic properties, improved AD-related neuropathological and cognitive deficits in SAMP8 mice, a genetic mouse model of sporadic AD (Adler et al., 2014). Importantly, there is also initial evidence suggesting that elders with mild cognitive impairment or established AD have lower concentrations of plasma amylin compared to age-matched healthy control subjects (Adler et al., 2014; Qiu and Zhu, 2014).

Taken together, the findings by Adler et al. (2014) and Zhu et al. (2015) emphasize the possibility that blunted (rather than increased) levels of peripheral amylin may underlie the development of neurological and cognitive impairments typically seen in mild cognitive impairment or established AD. This pathological link may be explained by a competition between amylin and Aβ to bind to its receptor in the CNS. Amylin and Aβ peptide both bind to the CTR, which in the CNS is ubiquitously expressed and complexed with the receptor activity modifying protein 3 (RAMP3) (Götz et al., 2013). According to a recent hypothesis (Qiu and Zhu, 2014), increased binding of Aβ to the CTR-RAMP3 complex which corresponds to the amylin-3 receptor occurs under conditions of hypoamylinemia when amylin fails to compete with Aβ for binding to this receptor complex. This in turn may facilitate Aβ oligomerization and plaque formation in the CNS (Qiu and Zhu, 2014). When hypoamylinemic conditions are restored by exogenous administration of non-amyloidogenic amylin or its clinical analogs, non-aggregated amylin may prevent the binding of Aβ to the CTR-RAMP3, thereby reducing the potency of Aβ peptides to oligomerize (Qiu and Zhu, 2014). In addition, the augmentation of non-amyloidogenic amylin may facilitate the clearance of Aβ from the brain, probably through its effects on cerebral vasculature (Westfall and Curfman-Falvey, 1995; Edvinsson et al., 2001; Qiu and Zhu, 2014).

Whatever precise mechanisms involved, it appears that hypo- and hyper-amylinemic conditions can both be associated with and promote the pathological effects of amyloid formation in the CNS. Further investigations are clearly warranted to explore how alterations in peripheral amylin secretion are linked to central amyloid formation. In the course of these attempts, it is of primordial importance to take into account the exact experimental conditions and in particular the clear species differences regarding the innate propensity of amylin to from plaques. Hence, it is absolutely critical to distinguish between amyloidogenic and non-amyloidogenic forms of amylin; the former is the case for human, other primates' and feline amylin, the latter for rodents (Betsholtz et al., 1989; Höppener et al., 1994; Moriarty and Raleigh, 1999; Matveyenko and Butler, 2006).

Another important factor that needs careful consideration is the clinical course of metabolic disturbances across aging, which in turn may critically determine the nature of peripheral amylin pathologies. As stated above, individuals with obesity or pre-diabetic insulin resistance often show signs of hyperamylinemia that precede the onset of full-blown T2DM (Johnson et al., 1989; Enoki et al., 1992; Westermark et al., 2011). Chronic over-production of amylin during these early stages of T2DM are readily toxic to pancreatic β-cells and eventually lead to β-cell loss (Höppener and Lips, 2006; Jurgens et al., 2011; Desai et al., 2014). As a consequence of the latter, the production and secretion of amylin may be severely impaired, thus leading to states of hypoamylinemia during late stages of T2DM (Qiu et al., 2014). Hence, both hyper- and hypo-amylinemia likely play a role in metabolic diseases such as T2DM depending on the clinical course and duration of the disease (Zhang et al., 2014).

**Figure 1:**
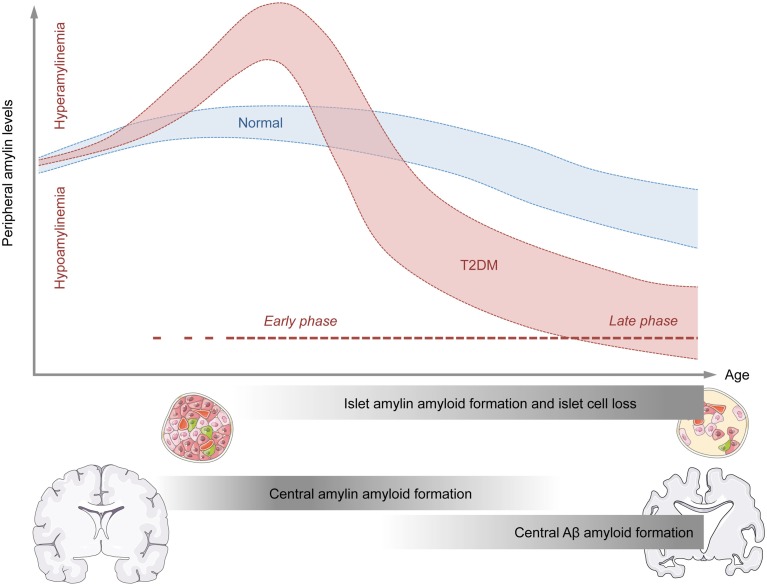
**Model for the temporal association between abnormal amylin production and peripheral and central pathologies relevant to neurodegenerative disorders**. The graphical illustration shows the putative relationship between age (x-axis) and peripheral amylin levels (y-axis) under normal conditions (blue) and conditions of type 2 diabetes mellitus (T2DM; red). The early phase of T2DM is typically associated with increased peripheral amylin (and insulin, not shown) secretion. Chronic over-production of amylin during the early stages of T2DM may facilitate amylin amyloid formation in pancreatic islet cells and eventually promote islet cell loss. As a consequence of the latter, the production and secretion of amylin (and insulin, not shown) may be severely impaired, thus leading to states of hypoamylinemia during late stages of T2DM. The initial hyperamylinemic condition occurring during the early phase of T2DM may further lead to amylin amyloid formation in the brain, which in turn may facilitate central Aβ amyloid formation and associated neurodegenerative processes during the late phase of T2DM.

The consideration of these temporal changes in amylin production may be important for attempts to explain why both hypo- and hyper-amylinemic conditions have been associated with increased amyloid formation in the CNS. On speculative ground, hyperamylinemic conditions may be more relevant for early processes of amyloid formation in the CNS, whereas hypoamylinemic conditions may be more strongly associated with late stages of central amyloid pathologies (Figure 1). The former conditions could indeed offer an explanation why amylin deposits can be present in brain parenchyma of AD patients who do not suffer from full-blown T2DM (Jackson et al., 2013), whereas the latter condition could explain why augmentation of non-amyloidogenic amylin can exert beneficial effects on amyloid clearance from the CNS once marked amyloids had already been established (Adler et al., 2014; Zhu et al., 2015).

## Concluding remarks

Epidemiological studies have established a clear association between metabolic and neurodegenerative disorders in general (Kleinridders et al., 2015), and between T2DM and AD in particular (Ott et al., 1996; Li and Hölscher, 2007; Butterfield et al., 2014; Desai et al., 2014). Amylin seems to be an important player at the interface between these metabolic and neurodegenerative disorders for several reasons. First, abnormal amylin production is a hallmark peripheral pathology both in the early (pre-diabetic) and late phases of T2DM, where hyperamylinemic (early phase) and hypoamylinemic (late phase) conditions coincide with hyper- and hypo-insulinemia, respectively (Figure 1). Second, there are notable biochemical similarities between amylin and Aβ, which (in some but not all species) are prone to self-aggregation and amyloid formation. The propensity of amylin to form amyloid plaques is not restricted to the peripheral organs such as pancreatic islet cells, but readily extends to the CNS, where is has been found to co-localize with Aβ plaques in at least a subset of AD patients. Hence, amylin may constitute a “second amyloid” relevant for the etiopathogenesis of AD and related neurodegenerative disorders (Jackson et al., 2013). In T2DM, the precise relationship between altered peripheral amylin production and central amylin amyloid formation appears complex and may be critically dependent on the temporal course of progressive pancreatic β-cell dysfunctions: Hyperamylinemic conditions may be more relevant for the early processes of amyloid formation in the CNS, whereas hypoamylinemic conditions may be more strongly associated with late stages of central amyloid pathologies. Normalization of hyperamylinemia during the early phase of T2DM may attenuate of even prevent subsequent amylin amyloid formation in the CNS, thereby exerting beneficial effects on progressive neurodegenerative disorders such as AD. On the other hand, augmentation of non-amyloidogenic amylin during late phases of T2DM, which are characterized by hypoamylinemic conditions, may offer a therapeutic strategy to facilitate amyloid clearance from the CNS and to block ongoing neurodegenerative processes once marked amyloids are established. More in-depth knowledge about these temporal relationships seems highly desirable as it may optimize the efficacy of amylin-based interventions in the treatment of AD and related neurodegenerative disorders with metabolic comorbidities.

### Conflict of interest statement

The authors declare that the research was conducted in the absence of any commercial or financial relationships that could be construed as a potential conflict of interest.
